# Privacy-Preserving IoT Data Aggregation Based on Blockchain and Homomorphic Encryption

**DOI:** 10.3390/s21072452

**Published:** 2021-04-02

**Authors:** Faiza Loukil, Chirine Ghedira-Guegan, Khouloud Boukadi, Aïcha-Nabila Benharkat

**Affiliations:** 1 University of Lyon, University Jean Moulin Lyon 3, CNRS, LIRIS, 69372 Lyon, France; 2 University of Lyon, iaelyon School of Management, University Jean Moulin Lyon 3, CNRS, LIRIS, 69372 Lyon, France; chirine.ghedira-guegan@univ-lyon3.fr; 3 Miracl Laboratory, Sfax University, 3018 Sfax, Tunisia; khouloud.boukadi@fsegs.usf.tn; 4 University of Lyon, INSALyon, CNRS, LIRIS, 69621 Lyon, France; nabila.benharkat@insa-lyon.fr

**Keywords:** privacy, Internet of Things, data aggregation, blockchain technology, homomorphic encryption technology

## Abstract

Data analytics based on the produced data from the Internet of Things (IoT) devices is expected to improve the individuals’ quality of life. However, ensuring security and privacy in the IoT data aggregation process is a non-trivial task. Generally, the IoT data aggregation process is based on centralized servers. Yet, in the case of distributed approaches, it is difficult to coordinate several untrustworthy parties. Fortunately, the blockchain may provide decentralization while overcoming the trust problem. Consequently, blockchain-based IoT data aggregation may become a reasonable choice for the design of a privacy-preserving system. To this end, we propose PrivDA, a Privacy-preserving IoT Data Aggregation scheme based on the blockchain and homomorphic encryption technologies. In the proposed system, each data consumer can create a smart contract and publish both terms of service and requested IoT data. Thus, the smart contract puts together into one group potential data producers that can answer the consumer’s request and chooses one aggregator, the role of which is to compute the group requested result using homomorphic computations. Therefore, group-level aggregation obfuscates IoT data, which complicates sensitive information inference from a single IoT device. Finally, we deploy the proposal on a private Ethereum blockchain and give the performance evaluation.

## 1. Introduction

Internet of Things (IoT) has emerged as one of the most significant technology in recent years. Its wide deployment has improved the quality of the individual’s lifestyle by providing better facilities on various daily applications, such as smart home, smart grid, and smart city. The IoT’s benefit to individuals’ lives is realized thanks to the analytics and aggregate information from the smart devices and the huge volumes of produced IoT data. However, the privacy-intrusive characteristic of the IoT technologies can discourage the citizens to participate in the IoT data analytics that may disclose their privacy. While aggregating IoT data can enhance decision-making processes, ensuring security and privacy in the data aggregation process is a non-trivial task. For instance, smart meter data aggregation can help citizens in a region to efficiently use energy; however, the single user’s electricity consumption data contains user-specific behavior patterns (e.g., presence at home), which may cause serious results once revealed.

Although several researchers have studied the privacy-preserving issue in the IoT data aggregation field, many challenges remain to be addressed: (i) the single point of trust on the data consumer, (ii) the raw data disclosure for the data aggregator, and (iii) the connection between the user’s identity and the used pseudonym in the IoT network. Finally, to our best knowledge, none of the existing proposals took the whole IoT data lifecycle, from the smart device owner’s consent to the IoT data analysis.

The first challenge is to send all the IoT data to a centralized structure that collects, stores, and processes these data. However, the centralized solutions suffer from the single point of trust issue. Indeed, the stored data have a risk to be modified or deleted by the centralized structure. Thus, this issue can affect the data aggregation result reliability. Moreover, the users (i.e., IoT data owners) have a limited control over how their data are handled by the data consumers.

In this context, multiple distributed approaches have been proposed to tackle the data aggregation issue with centralized solutions. Therefore, several users can collaborate with one another and aggregate their IoT data before sending the obtained result to the consumer. Thus, distributed approaches select one data aggregator per cluster or form an aggregation tree where each node aggregates the received data from its children. Although the transmitted data are encrypted, the distributed approaches might face the challenge of raw data disclosure. Indeed, the data aggregator needs to decrypt all the IoT data to aggregate them. However, the risk of cyber-attacks arises, and real-time data can be eavesdropped by hacking into the aggregator node and illegitimately used. To tackle this issue, existing distributed approaches used the homomorphic encryption technology.

The third challenge is to hide the connection between a real identity and the used pseudonym in an IoT network. Pseudonymity can be used to disguise the user’s identity. However, this connection may be disclosed by matching the individuals’ profiles with their behaviors.

Motivated by the above drawbacks, we focus on ensuring tamperproof communication, group-level aggregation, and pseudonymity to preserve privacy driven by blockchain and smart contracts during the data aggregation process. We look at how to enforce these privacy requirements in an end-to-end IoT data aggregation scheme. The latter ensures the computation over encrypted data without decrypting them and, therefore, compromised involved parties cannot access raw IoT data. The objective of this work is to propose PrivDA, a Privacy-preserving IoT Data Aggregation scheme. PrivDA takes advantages from both the blockchain and homomorphic encryption technologies, constituting an original contribution. In this work, we take advantages of the blockchain technology by using it as (i) a distributed data storage that eliminates the single point of trust issue of centralized storage solutions, (ii) a decentralized tamperproof communication history that facilitates coordination between several untrustworthy parties in the IoT domain, and (iii) a data aggregation controller without relying on a trusted aggregator using a smart contract. The latter is proposed and hosted on the blockchain to put several IoT devices in the same group based on the smart device owners’ privacy choices. We also take advantage of blockchain technology by using its self-executed programs, known as smart contracts. In our case, the proposed smart contract enables (i) the publishing of a data consumer’s request, (ii) the grouping of potential data producers (i.e., IoT devices) that can answer the request, and (iii) the publishing the request result. Furthermore, on top of the blockchain, we benefit from homomorphic encryption technology, which overcomes the raw data disclosure problem. By enabling computation over encrypted IoT data, the result can be computed without revealing the raw IoT data to the aggregator or the consumer. In this way, the proposed solution eliminates the need to trust a centralized consumer or a data aggregator while keeping the IoT data analysis accuracy. Finally, to tackle the third mentioned challenge, we allow each group member to create multiple pseudonyms and submit its IoT data under different pseudonyms.

The main contributions of this paper are summarized as follows:

1. We highlight the benefits of using the blockchain technology to facilitate coordination between several untrustworthy parties in the IoT domain and the homomorphic encryption technology to enable computation over encrypted data without revealing the raw data during the IoT data aggregation process and adopt them in a new scheme, called PrivDA.
2. We detail the privacy policy generation process that aims at matching between the data producers’ privacy preferences and the data consumers’ terms of service based on our semantic-based privacy-preserving API.
3. We introduce the group-level aggregation that aims at preserving privacy during the whole process of collecting, transmitting, storing, and processing IoT data in a decentralized network based on the proposed IoTDataAggregation smart contract.

This paper is organized as follows. Section 2 analyses existing solutions that studied the privacy-preserving issue in the IoT domain. Preliminaries are given in Section 3. Section 4 defines the proposed system model. Section 5 presents the proposed IoT data aggregation scheme. Security analysis and performance illustrated by experiments are detailed in Section 6 and Section 7, respectively. Finally, Section 8 concludes the paper and presents some future endeavors.

## 2. Related Work

The privacy-preserving data aggregation has become a research concern because it can guarantee the privacy of sensitive information during the data aggregation process. In fact, several solutions have been proposed to address this field in the IoT domain. Reading through the related work, we summarize the related proposals on three aspects focusing on (i) preserving the user’s privacy based on distributed data aggregation algorithms [1,2,3], (ii) protecting the user’s raw data based on the homomorphic encryption technology [4,5,6,7,8,9], and (iii) protecting the user’s identity based on the blockchain technology [10,11].

Distributed data aggregation was addressed by Rottondi et al. [1] who proposed a distributed aggregation of additive energy consumption metering data by relying on local gateways placed in households. These gateways collected the data generated by local smart meters and provided communication and cryptographic capabilities. However, this paper dœs not address the collision issue during the distributed aggregation. For their part, Corrigann-Gibbs and Boneh [2] have proposed Prio, a system that allowed a set of servers to compute aggregate statistics over client-provided data while maintaining client privacy and defending against client misbehavior. Recently, He et al. [3] proposed a consensus-based data aggregation algorithm that allowed neighbors to self-organize into several clusters. Data are aggregated in each cluster without revealing each node data to any other node (including the aggregator). Moreover, the aggregator in this model should have the knowledge of the topology of the overlay network that should be a connected, undirected graph. However, in the IoT domain, it is difficult to discover in advance the topology of the network due to its dynamism. In this context, the blockchain technology can be considered as a distributed paradigm that overcomes the two aforementioned issues by providing a decentralized tamperproof communication history that facilitates coordination between several untrustworthy parties in the IoT domain.

In order to overcome the raw data disclosure issue, several solutions [4,5,6,7,8,9] were proposed to use the homomorphic encryption technology to protect the user’s privacy while guarantying the data accuracy. For instance, smart metering is addressed by proposing an identity-based data aggregation protocol for smart grid to protect against unintentional errors and maliciously altered messages [4], a multi-service and user self-controllable smart metering system based on selective aggregation method [5], and a model in which trusted smart meter devices exchanging consumer’s measurements with the secured aggregator, performed inside an SGX enclave [6]. For the IoT domain, privacy-preserving data aggregation schemes have been proposed for mobile edge computing-assisted IoT applications [7] as well as for fog-enhanced IoT [8] based on both local certificate authority and the trusted certificate authority to generate the pseudonym certificates. Besides, Wang et al. [9] proposed an anonymous aggregation scheme in fog-based public cloud computing. Bilinear pairings and the Castagnos–Laguillaumie cryptosystem are used to enable computation over ciphertexts. These data aggregation-based schemes [4,5,6,7,8,9] aimed at guarantying the privacy of sensitive information between the data producer and the aggregator as well as saving bandwidth between the aggregator and the consumer. In the IoT domain, reducing the communication cost is important for proposing an efficient solution for physically constrained IoT devices. In fact, the blockchain technology can contribute to save more computing resources of the data aggregator by delegating the verification of data authentication, authorization, and integrity to a smart contract while eliminating trusted entity.

Through using blockchain technology, some other solutions [10,11] were tried to perform a secure data aggregation to protect the user’s identity. For instance, Guan et al. [10] proposed a privacy-preserving data aggregation scheme in a smart grid. Smart meters are divided into groups with each group having a private blockchain to record the participants’ data. For data aggregation, one smart meter is chosen to aggregate the group members’ data and record the aggregated data into the group’s private blockchain. For their part, Wang et al. [11] proposed a data aggregation framework to aggregate and verify meter data using a hierarchical blockchain system. Moreover, smart meters are grouped as clusters based on their geographical location. Each cluster is equipped with an aggregator that forwards the aggregated data to the substation. The first difference between our proposed scheme and the two blockchain-based proposals [10,11] is the used blockchain platform. In fact, the Merkle Tree blockchain system is used in [10], then the hierarchical blockchain system is adopted in [11], while the proposed scheme in this paper is based on the Ethereum blockchain. Besides, although the hierarchical blockchain system in [11] requires a blockchain platform with smart contract capability, it dœs not include the design or the use of smart contracts, contrary to what we propose in this paper. The reason behind proposing a new smart contract is to (i) enforce a common agreement between several untrusted parties without the involvement of a trusted third party; (ii) organize smart devices into groups according to their owners’ privacy choices; and (iii) prevent any identity fraud attempts concerning the smart devices, the aggregator, and the key generation authority.

## 3. Preliminaries

This section illustrates some notations used in this paper. Moreover, as mentioned above, the proposed solution is based on both blockchain and homomorphic encryption technologies, which are also introduced in this section.

### 3.1. Notations

Several notations are used in the next sections, thus Table 1 illustrates them with their descriptions.

### 3.2. Blockchain Technology

The blockchain technology is a distributed computing paradigm that successfully overcomes the problem related to the trust of a centralized party. Thus, in a blockchain network, several nodes collaborate among them to secure and maintain shared transaction records in a distributed way without relying on any trusted party. Specific nodes in the network known as miners are responsible for collecting transactions into blocks, solving challenging computational puzzles in order to reach consensus, and adding the blocks to a distributed public ledger known as the blockchain. Immutability is one of the essential characteristics of blockchain technology. The data stored on the blockchain are unchangeable records whose states cannot be modified after they are created. Immutability is interlinked with security, resilience, and irreversibility. Because of the transparency characteristic of public blockchains, it is possible to verify that the data of a transaction has existed at a specific time, but by keeping public keys anonymous, the identity of participants in real-life cannot be revealed. Thus, transactions are publicly accessible, but without information linking the transaction to anyone [12].

The first proposed system based on this technology was Bitcoin [12], which allows users to transfer securely the currency (bitcoins) without a centralized regulator. Ethereum [13] is another blockchain-based system that can also be used for the cryptocurrency. Unlike Bitcoin, Ethereum has the ability to use a smart contract, which is a common agreement between two or more parties. It stores information, processes inputs, and writes outputs thanks to its predefined functions [13].

In recent years, other researchers in [10,11] demonstrated how the blockchain technology can be used to address other domains, like the IoT data privacy-preserving.

### 3.3. Homomorphic Encryption Technology

The homomorphic encryption is a special encryption schema, in which some computation results can be obtained over ciphertext calculation without knowing the appropriate plaintexts and private keys of the ciphertexts [14]. Thus, an encryption scheme is called homomorphic over an algebraic operation, denoted as ⊕ only if $E(M_{1}⊕M_{2})$ can be computed from $E\left(M_{1}\right)⊕E\left(M_{2}\right)$, with E() is a homomorphic encryption function and $M_{1},M_{2}\in Z_{N}$ are two data items. There are several homomorphic encryption schemes in the literature, such as RSA [15], ElGamal [16], and Paillier cryptosystem [17]. According to the authors of [14], both RSA and ElGamal cryptosystems are only multiplicatively homomorphic. Therefore, they do not allow the homomorphic addition of ciphertexts. However, the Paillier cryptosystem implements the additive and multiplication operations. For this reason, we employ the Paillier cryptosystem in our study in order to address the privacy issue in the blockchain-based data aggregation process.

Principles of the Paillier cryptosystem [14]:

- **Key generation.** Let N=pq, where *p* and *q* are two large primes such that gcd(pq,(p−1)(q−1)) = 1, with gcd represents the greatest common divisor. Let λ=lcm(p−1,q−1), where lcm refers to the least common multiple. Then, select $g\in Z_{N^{2}}^{*}$, such that *N* satisfies the order divisible by *g*. Set function L(u) as L(u)=(u−1)/N and check the existence of μ = $L(g^{\lambda }$ mod $N^{2}{))}^{-1}\;\mathrm{mod}\;N$ to ensure *N* divides the order of *g*. Then, the public and private keys are generated as $Pk_{Pai}=\left(N,g\right)$ and $Sk_{Pai}=\left(\lambda \left(N\right),\mu \right)$, respectively.
- **Public key encryption.** For each message $M\in Z_{N}$, the number $r\in Z_{N}^{*}$ is randomly chosen and *M* is encrypted as follows:
  (1) $$C=Enc\left(M,Pk_{Pai}\right)=g^{M}r^{N}\;\mathrm{mod}\;N^{2}$$
- **Private key decryption.** Let a ciphertext $C\in Z_{N^{2}}^{*}$, the decryption is done by
  (2) $$M=Dec\left(C,Sk_{Pai}\right)=\frac{L\left(C^{\lambda }\;\mathrm{mod}\;N^{2}\right)}{L\left(g^{\lambda }\;\mathrm{mod}\;N^{2}\right)}\;\mathrm{mod}\;N$$
- **Evaluation.** Let $Enc(M_{1},Pk_{Pai})$ and $Enc(M_{2},Pk_{Pai})$ be two encrypted messages and $M_{1},M_{2}\in Z_{N}$, the ciphertext is calculated as follows:
  (3) $$\begin{array}{cc} Enc\left(M_{1},Pk_{Pai}\right).Enc\left(M_{2},Pk_{Pai}\right)\; & =g^{M_{1}}r_{1}^{N}g^{M_{2}}r_{2}^{N}\;\mathrm{mod}\;N^{2}\\ \; & =g^{M_{1}+M_{2}}{\left(r_{1}r_{2}\right)}^{N}\;\mathrm{mod}\;N^{2}\\ \; & =Enc\left(M_{1}+M_{2},Pk_{Pai}\right) \end{array}$$
  As a result, without knowing the plaintexts of $M_{1}$ and $M_{2}$, the encrypted value of $M_{1}+M_{2}$ can be obtained. The private key holder can read the plaintext sum by using the decryption function.

After introducing some notations and technical backgrounds used in the next sections, we define our proposal, which is a privacy-preserving IoT data aggregation scheme based on both blockchain and homomorphic encryption technologies. In the following sections, two parts are discussed: the system model and the basic scheme.

## 4. System Model

This section includes both the system model’s main goals and the system model’s description.

### 4.1. System Model Main Goals

Although multiple researchers have studied the IoT data aggregation field, many challenges remain to be addressed in order to tackle the privacy-preserving issue in this field. First, a distributed data storage is needed to eliminate the single point of trust problem that consists in storing, aggregating, and analyzing all the produced data by a centralized authority. Second, an end-to-end encryption data aggregation is needed to overcome the raw data disclosure issue that consists in giving all the raw data produced by the smart devices to a trusted party to be aggregated. Third, both anonymity and pseudonymity disguise the user’s identity; thus, they need both to be used to hide the connection between the real identity and the used pseudonyms (i.e., blockchain address) in the IoT network. Note that the blockchain address is considered as anonymous information according to existing privacy regulations, such as GDPR [18] that defines blockchain public key/address as data that “can no longer be attributed to a specific data subject”, and are thus pseudonymous data according to Article 4(5) GDPR [19]. Finally, a privacy-preserving solution needs to take advantage of the asymmetric encryption, the hash functions, and the digital signature in order to guarantee the three security properties: the data confidentiality, the data integrity, and the sender’s identity checking (i.e., authentication data). To the best of our knowledge, none of the existing solutions considered all the privacy requirements mentioned above, while covering the whole IoT data lifecycle, from the user’s consent to the data analysis. For this purpose, we propose PrivDA, an end-to-end privacy-preserving IoT data aggregation solution based on both blockchain and homomorphic encryption technologies. Indeed, our solution guarantees three security properties, namely data confidentiality, data integrity, and sender’s identity checking and two privacy properties: anonymity and pseudonymity.

### 4.2. System Model Description

In order to provide better facilities for the users, the consumers (e.g., energy substation as used later in our use case section) need to collect and analyze the produced data from the smart devices. However, IoT data analytics increases the user’s worries about the potential uses of collected data. In fact, the users desire to preserve their privacy while taking advantage of the offered services. Thus, collaboration between several users to aggregate IoT data prevents the consumer from learning individual data. However, aggregating IoT data requires a network manager and a trusted aggregator. We aim at addressing this dilemma by introducing a system model that improves the users’ privacy while keeping the data accuracy. Thus, our system model is based on (i) the blockchain technology that acts as a distributed data storage, (ii) the smart contract that acts as a data aggregation controller, and (iii) the homomorphic encryption technology that enables the computation over encrypted data without trusting a consumer or an aggregator.

**Figure 1:**
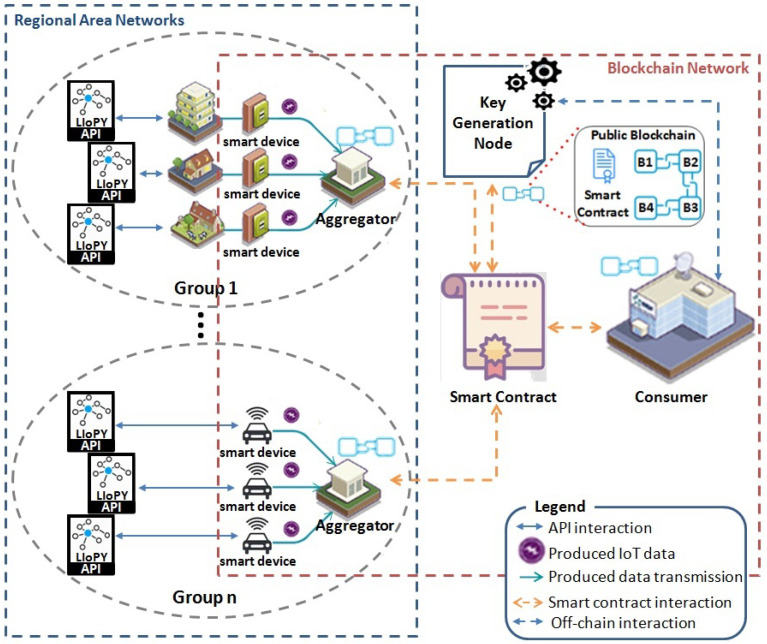
PrivDA System Model Overview. It is a smart space network that consists of two networks: regional area and blockchain networks. The regional area network includes several groups, each of which consists of a set of smart devices and one data aggregator. These groups are connected with the blockchain network thanks to a smart contract created by one consumer in order to receive some aggregated data as a request result. The blockchain network includes several nodes: a smart device, an aggregator, a consumer, and a key generation node. Each node has at least one blockchain address to interact with the blockchain, which is a distributed tamperproof communication history data storage.

As depicted in Figure 1, the system model consists of seven components: semantic-based privacy-preserving API, smart device, aggregator, group, smart contract, key generation node, and consumer.

The detailed description of these components is as follows.

- **Semantic-based privacy-preserving API**: It is a semantic engine that matches the consumer’s terms of service and the user’s privacy preferences to generate a common privacy policy that reflects the user’s privacy choices. This API is based on an IoT privacy ontology, called LIoPY, which is a European Legal compliant ontology that supports preserving IoT PrivacY and which has been defined in our previous work [20]. Thanks to LIoPY use and according to Algorithm 1, a privacy policy can be inferred. A policy is a set of conditions that the consumer needs to fulfill in order to handle specific shared IoT data. As demonstrated in our previous work [21], when hosting these conditions in a smart contract, privacy violation attempts can be prevented and the shared data will be handled as expected. Therefore, according to the user’s privacy preferences, a privacy policy is generated for each smart device.
- **Smart device**: It is an IoT device equipped with sensing and communication capabilities for collecting the produced data, encrypting them with both $Pk_{Pai}$ and $Pk_{Agg}$ keys, and storing them in smart contract. Group-level aggregation and computation over encrypted data are ensured by an untrustworthy aggregator.
- **Aggregator**: It is a smart device with high memory and storage capabilities, and it acts as a relay between the smart devices and the consumer through the latter’s smart contract. It records the aggregation result into the smart contract by updating the request result value after aggregating all the members’ data of a group.
- **Group**: It is a set of smart devices with one selected aggregator which is intended to obfuscate the individual IoT data of each of group members by computing an aggregated result from the members’ data. Groups are formed based on the members’ privacy choices, which are stored on the consumer’s smart contract.
- **Smart contract**: It aims at organizing several producers into groups based on their privacy preferences to collaborate among them and send only aggregated IoT data as a request result to the consumer, which is the owner of the smart contract. Indeed, group-level aggregation prevents the consumer to learn individual data. In order to eliminate a trusted aggregator, a public key based on the Paillier cryptosystem [17] is stored in the contract once generated by a key generation node.
- **Key generation node**: It ensures blockchain and off-chain computations by (i) generating pair of keys using Paillier cryptosystem [17] off-chain to enable additive computation over encrypted data, (ii) uploading the obtained public key $Pk_{Pai}$ on the smart contract using web3.js library [22], and then (iii) sending the corresponding secret key $Sk_{Pai}$ to the consumer off-chain. First, it computes a pair of keys using the Paillier cryptosystem. The latter executes the key generation function, then returns back the result to the key generation node. The latter updates the group public key by invoking a smart contract function and finally shares the group secret key with the consumer off-chain. This node is honest-but-curious; thus, it follows protocol specifications but may try to find out confidential data. Although this node generates the Paillier cryptosystem pair of keys, it cannot recover the plaintext of the stored IoT data in the smart contract because they are not only encrypted using the public key $Pk_{Pai}$, but also encrypted using the aggregator’s public key $Pk_{Agg}$. Thus, the need to trust the key generation node is eliminated.
- **Consumer**: The consumer can be an energy substation, a traffic routing station, or a scientific researcher who needs IoT data for an analytic purpose. A consumer creates a smart contract while indicating a key generation node blockchain address. Once hosted on the blockchain, the consumer can publish its terms of service, create an IoT data request, and receive the encrypted request result. Then, it uses the group’s secret key to decrypt the result.

After defining the system model’s core components, we detail below our proposed privacy-preserving IoT data aggregation scheme.
**Algorithm 1** Privacy Attribute Matching    **Input:** Terms_of_Service ToS    **Output:** Privacy_Policy PPolicy1:PRule←⌀; PPolicy←⌀2:DCategory=ToS.requestedData.hasDataCategory3:PRule=DCategory.hasPrivacyRule4:intendedPA = {Purpose, Operation, Disclosure, Retention, Condition}5:**for each**ipa in intendedPA **do**6: **switch** (ipa)7: **case** Purpose:8:  **if** (ToS.requestedPurpose ∉ ProhibitedPurposes **and** ToS.requestedPurpose ∈ AllowedPurposes) **then**9:   
PPolicy.effPurp=ToS.requestedPurpose
10:  
**else**
11:   return PPolicy12:  
**end if**
13: **case** Operation:14:  **if** (ToS.requestedOperation ∉ ProhibitedOperations **and** ToS.requestedOperation ∈ AllowedOperations) **then**15:   
PPolicy.effOpe=ToS.requestedOperation
16:  
**else**
17:   return PPolicy18:  
**end if**
19: **case** Disclosure:20:  **if** (ToS.requestedDisclosure ∉ ProhibitedDisclosures **and** ToS.requestedDisclosure ∈ AllowedDisclosures) **then**21:   
PPolicy.effDisc=ToS.requestedDisclosure
22:  
**else**
23:   return PPolicy24:  
**end if**
25: **case** Retention:26:  **if** (ToS.requestedRetention<PRule.retention) **then**27:   
PPolicy.effRet=ToS.requestedRetention
28:  
**else**
29:   return PPolicy30:  
**end if**
31: **case** Condition:32:  **if** (PRule.icond.allowedRole == ToS.hasInitiator.hasRole) **then**33:   
PPolicy.effCondition=PRule.icond
34:  
**else**
35:   return PPolicy36:  
**end if**
37: 
**end switch**
38:**end for**39:PPolicy.hasRequestedOutput= output40:PPolicy.hasAccessDecision= Permit41:PPolicy.hasPrivacyObligation=PRule.hasPrivacyRuleObligation42:**return**PPolicy


## 5. PrivDA: A Privacy-Preserving IoT Data Aggregation Scheme

In order to detail the proposed scheme, we discuss below seven phases: system initialization, privacy policy generation, data collection, data transmission, data verification, data aggregation, and data aggregated reading.

### 5.1. System Initialization

This first phase consists in (i) deploying an instance of the IoTDataAggregation smart contract by a consumer, (ii) grouping the data producers based on their privacy policies, and (iii) sharing the group keys generated by the key generation node with the data producers and the consumer.

The proposed IoTDataAggregation smart contract is designed for enabling a data consumer to publish a data request, a key generation node to share a Paillier cryptosystem public key per group, a data producer to participate into group-level aggregation to answer request, a data aggregator to verify the data of the group members before aggregation, and finally update the request result that can be retrieved by the consumer and decrypted with the appropriate group’s secret key off-chain. Thus, it is deployed by one consumer that wants to receive aggregated data as a request’s result from a group of data producers (i.e., smart devices). Thus, the consumer sends a transaction that invokes the IoTDataAggregation smart contract constructor to deploy it on the blockchain while indicating the blockchain address of the key generation node. The blockchain address of the key generation node must be set when deploying/hosting the smart contract and remains immutable; thus, it cannot be replaced.

Once the smart contract is hosted on the blockchain, the entire blockchain network can interact with it by invoking its predefined functions. Indeed, the consumer chooses the requested IoT data and defines its terms of service by invoking the defined *updateToS* function. After that, the smart contract enables the data producers to define the privacy choices of their owners. Thus, based on these privacy choices, the smart contract can decide whether or not one producer will be included in a specific group. According to the number of each group’s members and the aggregator’s capacity, an aggregator is selected to aggregate the data of all the group’s members and update the request result on the smart contract.

Finally, once the group is created, the producers are added, and the aggregator is selected; the key generation node needs to compute the Paillier cryptosystem pair of keys off-chain and then upload the public key on the smart contract by invoking the *updatePK* function. This function can only be invoked by the key generation node’s blockchain address. The secret key $Sk_{Pai}$ is only shared with the consumer (i.e., the smart contract owner) off-chain through a secured channel.

To sum up, the smart contract puts together into one group potential data producers that can answer the consumer’s request and chooses one aggregator the role of which is to compute the group requested result using homomorphic computations. Due to the lack of space, we provide the full definition of the smart contract at our Github-repository (https://github.com/Floukil/E2EAggregation/blob/master/IoTDataAgg (accessed on 30 March 2021)).

### 5.2. Privacy Policy Generation

**Figure 2:**
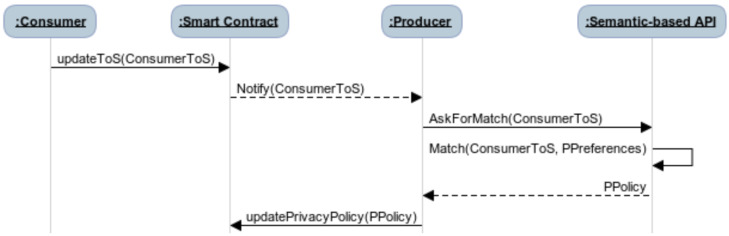
The process for privacy policy generation.

This phase combines blockchain and off-chain semantic computations to generate and share a privacy policy between data consumer and producer. First, the consumer uploads its terms of service by invoking the *updateToS* function defined in the smart contract. Once this function event is emitted, each producer computes a new privacy policy using the semantic-based privacy-preserving API. The latter matches the terms of service and the user’s privacy preferences, then returns back the result to the producer that uploads the generated privacy policy on the smart contract by invoking the *updatePrivacyPolicy* smart contract function. Figure 2 depicts the process of generating a new privacy policy between a data producer and a data consumer using the proposed IoTDataAggregation smart contract and the semantic-based privacy-preserving API.

As mentioned above, the API is based on the Algorithm 1 which uses LIoPY concepts to generate a privacy policy. Moreover, Algorithm 1 takes as input the consumer’s terms of service and returns an instantiated privacy policy if there is a match with the appropriate data category’s privacy rule of the requested data. LIoPY [20] considers a set of privacy attributes: Consent, Purpose, Retention, Operation, Condition, and Disclosure. These attributes specify, respectively, the user’s awareness, for what reason, for how long, how, under which conditions the user’s data will be handled, and to whom they can be disclosed. According to each privacy attribute, a matching type is defined. For instance, according to the European General Data Protection Regulation (GDPR) [18], without explicit acceptance of the user, personal data should not be disclosed to third parties. Thus, when an IoT data item (e.g., smart meter data) is requested, the required disclosure ToS.requestedDisclosure is checked against the intended allowed disclosures AllowedDisclosures and the intended prohibited disclosures ProhibitedDisclosures according to the privacy rule of the output category PRule (Line 20). Thus, the requested disclosure should belong to the set of the allowed disclosures and not to the set of the prohibited disclosure defined by the user. On the other hand, a privacy policy is created only if all the privacy rule’s attributes match all the privacy attributes of the terms of service. A set of effective privacy attributes is associated with the generated privacy policy. Moreover, some privacy obligations are associated with the privacy policy according to the data category’s privacy rule of the requested data (Line 41). Thus, the generated privacy policy defines how the data can be handled by the consumer once shared. Once generated, the policy is published on the contract as an agreement with the consumer, that is considered as an untrusted party. The reason behind storing the privacy policy on the contract has a twofold benefit: First, it can enforce privacy policy compliance by preventing any privacy violation attempt without the involvement of a trusted third party. Indeed, before adding any data producer to a specific group, the contract checks the requested data against the allowed and prohibited data items that are defined on the data producer policy. Second, it improves the non-repudiation principal compliance, which consists in preventing any IoT network node from denying actions that are performed by itself. Indeed, the producers’ owners cannot deny having sent their consent to use their data. Nevertheless, they can revoke the granted privacy permissions for a consumer by updating their consent to Deny instead of Permit.

### 5.3. Data Collection

**Figure 3:**
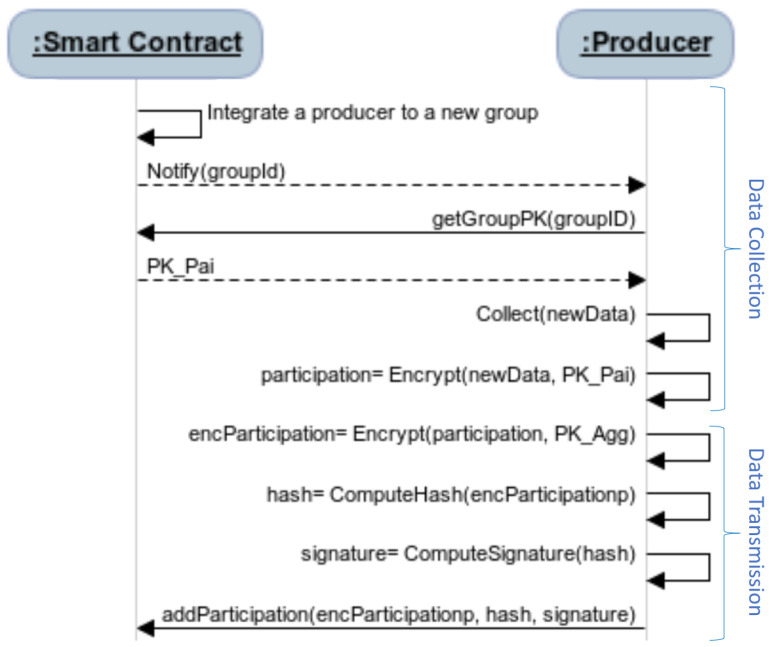
The process of computing participation during the data collection phase and before the data transmission.

Figure 3 depicts the process of computing the participation of a data producer during both collection and transmission phases. Based on the stored privacy policies, the smart contract decides whether or not one smart device is included in the created group. In case of an inclusion, each group member collects its produced data according to the requested data in the appropriate group. Then, it retrieves the group public key of the Paillier cryptosystem [17] published on the smart contract by the key generation node and encrypts the data, denoted as participation using the public key $Pk_{Pai}=\left(N,g\right)$.

Both the encrypted collected data participation and the public key $Pk_{Pai}$ are the main elements used in the rest of the IoT data lifecycle, namely, the data transmission, verification, and aggregation, until data aggregated reading.

### 5.4. Data Transmission

In order to secure the data during the transmission phase, encryption, hash, and signature techniques are applied.

To guarantee the data confidentiality, the selected aggregator shares a public key, denoted as $Pk_{Agg}$, with the group’s members and keeps the corresponding private key, denoted as $Sk_{Agg}$ secret. Each group’s member encrypts off-chain the collected data with the aggregator’s public key and gets the encParticipation, as depicted in Figure 3.

Moreover, the hash functionality and the digital signature are used in order to enable the message receiver (i.e., the selected aggregator) to check the data integrity and verify the sender’s identity. Each participant of the blockchain network has at least a blockchain address with one related pair of keys, a public key $Pk_{SD}$, which is shared by all the participants and a private key $Sk_{SD}$, which is kept secret by each participant.

Thus, the hash is calculated from the encrypted produced data, which is denoted as encParticipation using the Secure Hash Algorithm SHA-2(256) [23] and the digital signature, which is denoted as signature, refers to the ciphertext of the digest produced by the sender’s private key $Sk_{SD}$.

Both the data hash hash and the digital signature signature are sent with the encrypted data encParticipation to be stored on the smart contract. Encrypting the collected data by both $Pk_{Pai}$ and then by $Pk_{Agg}$ prevents the key generation node and the aggregator to learn plaintext. Besides, the smart contract offers two verification functionalities, which consist of the hash value verification and the signature verification.

### 5.5. Data Verification

**Figure 4:**
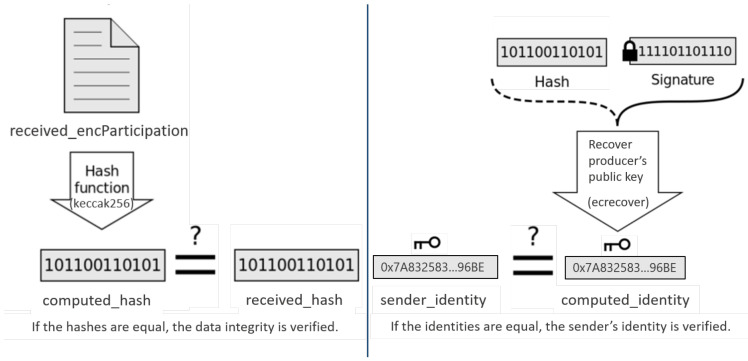
Verification of data integrity and sender’s identity.

To verify the transmitted data, the smart contract computes the computed_hash, which is the hash of the received encrypted data, denoted as received_encParticipation using the hash function keccak256, as depicted in Figure 4. In case of an equality between both the received hash hash and the internally computed hash computed_hash by the *verifyHashVal* contract function, the smart device participation is accepted and added to the group’s participation list.

Moreover, the smart contract enables the aggregator to verify the smart device identity, denoted as sender_identity, which presents the sender’s blockchain address. Therefore, the signature verification function, denoted as ecrecover, recovers the blockchain address using the hash of the encrypted data and its signature, as depicted in Figure 4.

The computed_identity is equal to the sender identity only if the same private key is used to sign the hash data.

The data verification functionality of PrivDA is offered by the proposed *verifyHashVal* smart contract function.

### 5.6. Data Aggregation

When the selected aggregator retrieves the participation of all the group members, it verifies the sender’s identity and the data integrity in order to prevent illegal smart devices sending malicious data. We assume that an attacker can eavesdrop the collected data by smart devices but cannot modify them, and therefore collected data by legal devices are trusted. If the data are verified, the selected aggregator decrypts all the participation using its private key, which is denoted as $Sk_{Agg}$. After that, the aggregator computes the sum of all the data, denoted as $M_{i}$, with 1≤i≤k and *k* is the number of the group members as follows:

(4) $$\begin{array}{cc} encRequestResult & =Enc\left(\sum_{i=1}^{k}M_{i},Pk_{Pai}\right)=\prod_{i=1}^{k}Enc\left(M_{i},Pk_{Pai}\right)\\ \; & =\prod_{i=1}^{k}g^{M_{i}}r^{N}\;\mathrm{mod}\;N^{2}=g^{\sum_{i=1}^{k}M_{i}}{\left(\prod_{i=1}^{k}r_{i}\right)}^{N}\;\mathrm{mod}\;N^{2} \end{array}$$

After computing the aggregation data off-chain, the selected aggregator updates the appropriate group request result by sending a transaction that invokes the *updateRequestResult* smart contract function. When the request result is stored on the smart contract, the *requestResultUpdated* event is emitted.

Although the considered data aggregation functionality in this paper is the SUM function, other aggregation functions, such as average, minimum, and maximum, can be applied in the IoT context. As it has been shown that any homomorphic encryption is insecure against ciphertext-only attacks (COA) if they support comparison operation, our solution can consider the minimum and maximum aggregation functions by performing addition operation instead of comparison operation on the data encrypted with homomorphic encryption schemes as proposed in [24].

### 5.7. Data Aggregated Reading

Once the result is updated on the smart contract, the consumer retrieves the appropriate group request result, denoted as encRequestResult. Then, the consumer uses the private key $Sk_{Pai}=\left(\lambda \left(N\right),\mu \right)$ of the Paillier cryptosystem [17] to decrypt the request result such that

(5) $$\begin{array}{cc} request\_result & =Dec\left(\frac{L\left(encRequestResult^{\lambda }\;\mathrm{mod}\;N^{2}\right)}{L\left(g^{\lambda }\;\mathrm{mod}\;N^{2}\right)}\;\mathrm{mod}\;N,Sk_{Pai}\right)\\ \; & =\sum_{i=1}^{k}M_{i} \end{array}$$

After that, the consumer ends the group by invoking the *endGroup* smart contract function that updates the group state from *IN_PROGRESS* to *FINISHED*.

In this way, the consumer can read the sum of all the group members without knowing the individual IoT data.

## 6. Security and Privacy Analysis

After detailing our proposal, we highlight and analyze in this section both the security and privacy properties.

### 6.1. Anonymity and Pseudonymity

Hiding the connection between each group member and its owner’s real identity is one of the challenges addressed by the proposed scheme. Anonymity and pseudonymity are a common solution to disguise the user’s identity in the blockchain network. However, the connection between the real identity and the pseudonym may be disclosed by matching the individuals’ profiles with their behaviors over a particular period of time [10]. The proposed scheme overcomes this issue by giving each group member the possibility to create and submit the IoT data under different pseudonyms. As the group member randomly picked a pseudonym, which is a random string, it remains unconditionally anonymous while using such pseudonym.

### 6.2. Against Sybil Attacks

Although allowing the smart devices to use several pseudonyms could improve the device owner’s privacy, it can also raises the risk of Sybil attacks in which a node in a peer-to-peer network operates multiple identities to gain the majority of influence in the network to carry out illegal actions [25]. In our case, we prevent Sybil attacks by putting a cost for every identity that aims to join the network. Indeed, before joining any group, each new identity needs to update its privacy preferences in the smart contract. This cost is not expensive to prevent legal devices to create some pseudonyms but can prevent malicious entities to create thousands of pseudonyms and control the entire system. Furthermore, enabling validation of new joiner identities by already established member’s group in the smart contract can be investigated in the future to improve Sybil-resistance.

### 6.3. Against External Attacks

External attackers can eavesdrop on the communication channels to get unauthorized IoT data. By encrypting all the IoT data by both the public key of the Paillier cryptosystem and the selected aggregator’s public key, PrivDA provides powerful protection against such attacks. Moreover, external attackers cannot alter transmitted data because they do not have the private key of devices to sign the hash of the encrypted data.

### 6.4. Data Confidentiality, Integrity, and Sender’S Identity

PrivDA can guarantee three security properties: data confidentiality, data integrity, and sender’s identity.

For data confidentiality, only a receiver with the appropriate private key can recover the encrypted message. Therefore, an adversary could eavesdrop the encrypted message, but without recovering the plaintext. We used the blockchain-based asymmetric encryption to guarantee the data confidentiality.

For the data integrity and the sender’s identity, the smart contract enables verifying the data integrity by comparing the received data hash and the computed hash. Moreover, any illegal smart devices can be detected by comparing the sender’s blockchain address to the recovered identity using the data hash and its digital signature. To ensure data integrity and realize a secure identity verification, we used the hash function and the blockchain-based digital signature, respectively. As such hash function and digital signature are provably secure, so are our data integrity and sender’s identity proofs.

To sum up, our scheme can ensure that each received message from the claimed sender can only be recovered by the intended receiver and has not been altered during the transmission process.

### 6.5. End-To-End Privacy-Preserving Solution

By enabling computation over encrypted IoT data, the result can be computed without revealing the raw IoT data to a consumer or a data aggregator. In this way, the need to trust the consumer or the data aggregator is eliminated during the collection, transmission, storage, and processing phases.

In data collection, the SD data are encrypted using a public key, which is denoted as $Pk_{Pai}$ of the Paillier cryptosystem [17]. The used public key is shared by all the group members while no one of the SDs has the corresponding private key, which is denoted as $Sk_{Pai}$ to recover the other ciphertexts. Moreover, each SD encrypts its generated ciphertext using the selected aggregator’s public key, denoted as $Pk_{Agg}$ before sending its data. Therefore, the plaintext cannot be known by an adversary that does not have both the aggregator’s private key $Sk_{Agg}$ and the private key $Sk_{Pai}$ even if it eavesdrops the ciphertext during the transmission phase. Although the consumer has the private key $Sk_{Pai}$, it cannot recover the plaintext because it does not have the aggregator’s private key $Sk_{Agg}$ to decrypt the message.

Moreover, in the data aggregation process, each group’s aggregator just computes the result over the received encrypted data without recovering the individual data of each SD. Thus, even if the aggregator is compromised, it cannot decrypt the ciphertexts because it does not have the appropriate Paillier cryptosystem private key $Sk_{Pai}$.

Last, when the consumer receives the computed result from the aggregator, it uses the Paillier cryptosystem private key $Sk_{Pai}$ in order to recover the final result of the aggregation process. Even if an adversary hacks into the consumer, only the sum of the aggregated data is exposed while SD’s individual data are not disclosed.

To sum up, the proposed scheme can ensure the SD’s data privacy during the whole IoT data lifecycle, namely, the collection, transmission, storage, and processing phases.

## 7. Experiments and Results

Ethereum is currently the most common blockchain platform for the development of smart contracts [13]. Therefore, we implemented our proposed smart contracts using the Solidity language [26] and deployed it to the Ethereum test network. Using the latter instead of the Ethereum real network has no impact on our system because it does not rely on the currency transfer. Thus, we created a test system using Truffle development framework [27], which is the most popular development framework for Ethereum. This framework, among others, generates JavaScript bindings for the smart contract, enables automated smart contract testing, and includes libraries such as web3.js [22] that facilitates the communication between the smart contract and the Ethereum clients. In our experiments, we used the contract events in order to automate the actions taken by the different nodes. Then, we implemented event callbacks in our testing framework using the web3.js library [22]. All the experiments were conducted on a computer with Intel Core i5 CPU (2.30 GHz and 8GB RAM).

### 7.1. Data Aggregation Use Case

We implemented a test system that consists of several nodes: one key generation node, one consumer, one aggregator, and 50 smart devices. We assumed that each node is represented by an Ethereum address associated with a pair of public/private keys.

**Figure 5:**
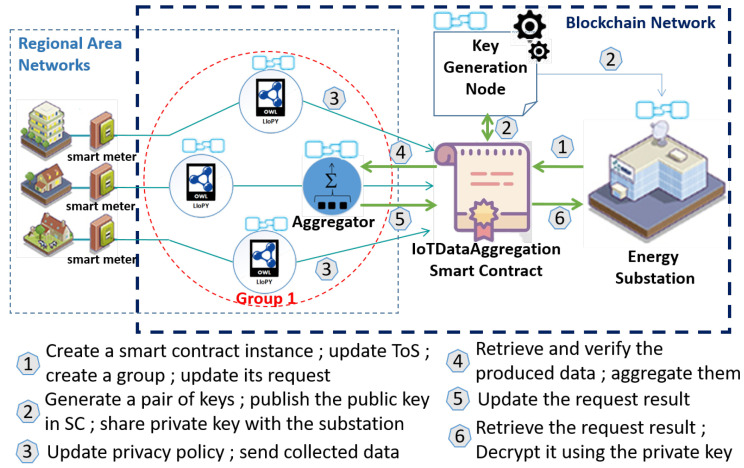
Use case description.

Let the smart devices be smart meters and the consumer an energy substation that asks for aggregated smart meter data every 15 minutes for a time duration of 30 days. Figure 5 depicts an example of our test system’s steps.

First, the energy substation creates a smart contract while indicating the blockchain address of a key generation node. The latter is a JavaScript node that supports the Paillier cryptosystem [17]. Second, the substation updates its terms of service by invoking the smart contract function, which is called *updateToS*. As mentioned above, the smart contract events are used to automate the actions taken by the different nodes. Thus, the smart meters update their privacy policies every time the terms of service are updated by invoking the smart contract function, which is called *updatePrivacyPolicy*. The privacy policy generation code is a JAR (Java ARchive) file, which is a package file format used to store many Java classes and associated metadata into one file for distribution. The used JAR is proposed based on Algorithm 1, which consists in matching the smart meter owner’s privacy preferences and the substation terms of service in order to generate a common privacy policy about sharing smart meter data. After that, the substation creates a group and publishes its request. Once the group is created, the key generation node generates off-chain a pair of keys ($Pk_{Pai}$, $Sk_{Pai}$), updates the group’s public key on the smart contract, and shares the private key with the substation. Based on the privacy policies, the smart contract decides whether or not one smart meter is included in the created group. Once the producers are added to the substation group, they periodically send their produced meter data. Meter data are assumed to be random numbers generated in the range of [0,4] kilowatt-hour (kWh). Then, the aggregator retrieves all the produced data, checks the integrity and the sender’s identity, aggregates them, and updates the request result by invoking the appropriate smart contract function. Once updated, the substation retrieves the request result and decrypts it using the private key $Sk_{Pai}$. When the retention duration ends, the substation’s group is automatically ended by invoking the *endGroup* function. To implement our use case, we deployed a smart contract and interacted with it by sending a set of transactions. During our experiments, we recorded the computing time, in milliseconds, of each aforementioned phase. Each phase consisted of one or several transactions that invoke the appropriate smart contract functions to read or write on the deployed smart contract.

In the rest of this section, our proposed scheme performance is evaluated in terms of computation complexity and cost. After that, we compare the proposal with some existing solutions in terms of communication cost and eavesdropping probability on private individual data.

### 7.2. Computation Complexity and Cost

We look into the computation complexity in the data processing, which includes three phases: data encryption, data aggregation, and data decryption. When the producer wants to send the IoT data, it encrypts the input with the Paillier encryption function, which needs two exponentiation operations in $Z_{N}^{*}$ and one multiplication operation. Besides, the data producer performs one hash operation in order to generate a digital signature that enables the verification of both the sender’s identity and the data integrity. When the data aggregator receives all the encrypted data from *k* producers, it verifies the validity of each sender’s identity and data integrity by performing *k* hash operations, then it computes the final result by multiplying all the received ciphertexts, which needs k+1 multiplication operations, then it executes one hash function before sending the result to the consumer. The latter executes one hash function to verify the received data, then decrypts the ciphertext with the Paillier decryption function, which needs one multiplication operation and one exponentiation operation in $Z_{N}^{*}$ in order to recover the plaintext.

Table 2 summarizes the computation complexity of the three scheme’s entities: the producer, the aggregator, and the consumer. For simplicity, the exponentiation operation is denoted as $C_{e}$, the multiplication operation is denoted as $C_{m}$, and the hash operation is denoted as $C_{h}$.

Moreover, to measure our solution’s performance, we conduct some experiments to deduce the appropriate number of group members that preserves each member’s privacy with a less computational cost. To this end, we evaluate whether the computing time of data aggregation is acceptable by making several tests using a different number of group members that increases from 5 to 50. Therefore, we perform a first experiment to measure the required time to check and compute the aggregated data result by an aggregator and a second experiment to measure the required time to decrypt the aggregated data result by a consumer.

**Figure 6:**
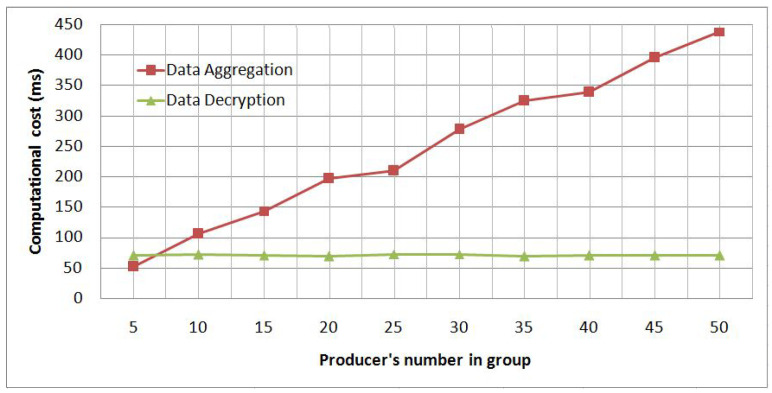
Computational cost of data aggregation and data decryption.

Figure 6 shows the computational cost of the data aggregation and decryption cases. The computational cost varies from 50 to 440 milliseconds(ms). We observe that data aggregation’s computational cost increases with the number of the group’s members, going from 51 to 440 ms. The cost increases linearly to reach 200 ms at 20 members, keeps the same level until 25 members, and then increases linearly again. This lets us conclude that the appropriate number of group members within a reasonable computational cost is between 20 and 25 members, whereas the data decryption computational cost is independent of the number of the group’s members because all the group’s members’ data are already aggregated by the aggregator. Therefore, the consumer receives one ciphertext that represents the sum of all the encrypted group’s data.

**Figure 7:**
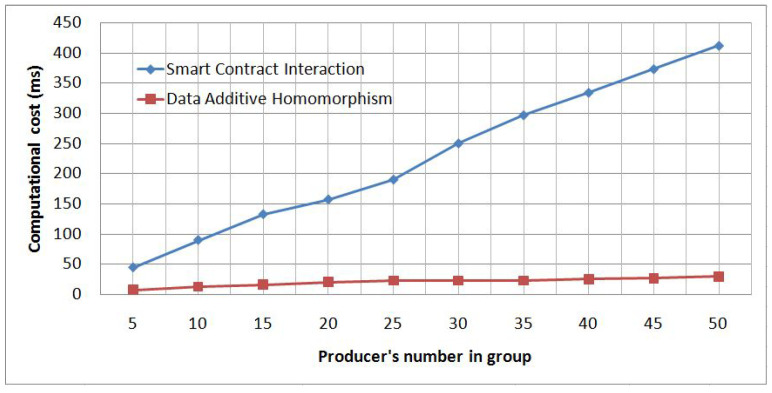
Data aggregation’s computational cost details.

In order to understand the data aggregation behavior, we split the data aggregation phase into two parts—the smart contract interaction and the data additive homomorphism—and conduct a new experiment to measure the required time for each part. As shown in Figure 7, the computational cost of the data additive homomorphism part varies only from 7 to 30 ms, as well as the computational cost of the smart contract interaction part varies from 45 to 412 ms, which explains the high data aggregation computational cost in our first experiment depicted in Figure 6.

Note that using a distributed system for storing and accessing data instead of storing all the group’s members’ data on the blockchain would be more appropriate in order to overcome the computational cost. Thus, the InterPlanetary File System (IPFS) [28] can be used to reduce both the smart contract interaction and the storage costs. IPFS is built on the top of both BitTorrent protocol [29] and the Kademlia DHT [30], which are well-known protocols for their ability to scale to a large number of nodes.

### 7.3. Communication Cost Comparison

The proposed scheme enables aggregating raw IoT data from several data producers into one ciphertext based on the Paillier cryptosystem [17]. In order to evaluate the efficiency of the proposed solution, we conduct an experiment to measure the communication cost from multiple producers to one aggregator, as well as from one aggregator to one consumer. After that, we compare the obtained communication cost by bits with three other related schemes [4,7,9] as summarized in Table 3.

In the proposed scheme, the ciphertext’s form is $C=g^{M}r^{N}\;\mathrm{mod}\;N^{2}$ and the bit length of *N* is |N|=1024. For the communication from *k* data producers to an aggregator, the communication cost is k×2048 bits because each data producer encrypts its data to one ciphertext, whose bit length is equal to $N^{2}$, i.e., 2048 bits. For the communication from an aggregator to a consumer, the overhead is independent of the data producer number because the data are aggregated by the aggregator before reaching the consumer. Therefore, the communication cost from an aggregator to a consumer is only 2048 bits. Therefore, the total communication cost is k×2048+2048 bits. Table 3 shows that our scheme consumes fewer bits than the three other related schemes [4,7,9] during the communication phase.

**Figure 8:**
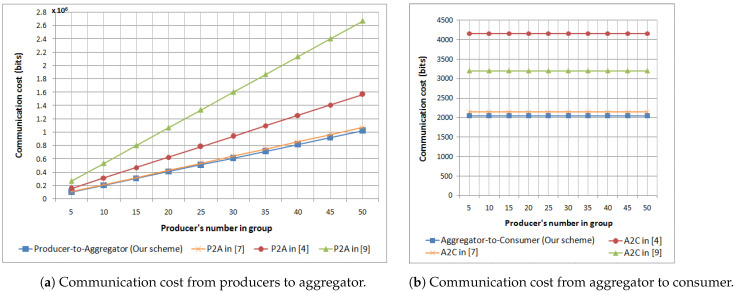
Communication cost comparison. The *x*-axis values are the number of data producers per one aggregator. Both *y*-axes represent the communication cost by bits, but with different measurement scale.

Figure 8 depicts the communication cost comparison of the considered schemes. From Figure 8a, we observe that the communication cost from multiple producers to one aggregator increases when we vary the number of data producers *k* from 5 to 50, while the communication cost from one aggregator to one consumer remains constant, as shown in Figure 8b. In both cases, the proposed scheme’s communication costs are less than the three other related schemes [4,7,9]. The difference between the proposed scheme and the other three schemes is the communicated information beside the ciphertext, such as the timestamp [7], the digital signature [4], and the authorization information [9]. In our case, such extra information is included in the blockchain’s transactions and verified by the smart contract before starting the communication with the data aggregator. Thus, the data aggregator dœs not need to deal with such verification. To sum up, Figure 8 clearly shows that the proposed scheme is more effective than the other schemes in terms of communication cost.

Nevertheless, we deduce that the producers do not need any additional computational capabilities to communicate with the aggregator as each producer sends one data item independently of the number of the group’s members. However, the more members the group includes, the greater the need for the memory and storage capabilities of the aggregator.

### 7.4. Comparative Capability Evaluation of Privacy-Preserving

We compare the overall probability of eavesdropping on private individual data in our proposal and the existing systems considering both a standard centralized system, where the consumer received all the producers’ data to aggregate them and the distributed system proposed in [11], where the consumer received only an aggregated result of all the producers’ data. We reuse the same conditions and variables provided in [11] to perform this comparison. Thus, we denote success probabilities of manipulating data in a consumer and obtaining its private key as γ and $\bar{\gamma }$, respectively. Similarly, we denote variables for both aggregator and producer as detailed in Table 4.

Table 5 shows the success probability of attackers to eavesdrop individual data by each data type considering three system types: centralized system, distributed system [11], and the proposed system. We do not consider eavesdropping when it only requires stealing the private key of the data sender. In Table 5, we denote producer as *P*, aggregator as *A*, consumer as *C*, and unconsidered eavesdropping probability as N/A.

For the centralized system, individual data can be eavesdropped by attackers through hacking an aggregator or a consumer node. Indeed, individual data are not encrypted in these nodes. Thus, the centralized system’s eavesdropping probability is equal to (β+γ)/2.

For the distributed system [11], individual data can be eavesdropped by attackers through simultaneously gaining the consumer’s private key and hacking into a data producer or a channel between data producers. Thus, the distributed system’s eavesdropping probability is equal to $(\bar{\gamma }\alpha +\bar{\gamma }\mu )/2$, with μ is the success probability of hacking into a communication channel and gain the sender’s private key, a data producer in this case, thus $\mu ≃\bar{\alpha }$, with $0<\bar{\alpha }<\alpha <1$.

For the proposed system, attackers can only eavesdrop individual data through simultaneously (i) gaining the private key of the consumer’s group and hacking into a data producer or (ii) stealing the private keys of both the consumer’s group and the aggregator and hacking into an aggregator. Thus, successful eavesdropping probability in this proposal is $(\bar{\gamma }\alpha +\bar{\gamma }\bar{\beta }\beta )/2$.

Let the consumer’s defensive capability stronger than the aggregator’s, and α is in the range of (0, 1); therefore, we deduce $\left(\bar{\gamma }\alpha +\bar{\gamma }\bar{\beta }\beta \right)/2<\left(\beta +\gamma \right)/2$, which means that the successful eavesdropping probability in the proposed system is less than the successful eavesdropping probability in the standard centralized system, as detailed below.

$$\left.\begin{array}{c} \bar{\gamma }<\gamma <\bar{\beta }<\beta \\ 0<\alpha <1 \end{array}\right\}\Rightarrow \bar{\gamma }\alpha <\gamma \Rightarrow \left(\bar{\gamma }\alpha +\bar{\gamma }\bar{\beta }\beta \right)/2<\left(\beta +\gamma \right)/2$$

Let the aggregator’s defensive capability be stronger than the data producer’s, where hacking into an aggregator is the precondition of gaining the node’s private key and hacking into a communication channel between producers is equal to gaining the producer’s private key; therefore,

$$\left.\begin{array}{c} \bar{\beta }<\beta <\bar{\alpha }\\ 0<\bar{\beta }<\beta <1\\ \mu ≃\bar{\alpha } \end{array}\right\}\Rightarrow \bar{\beta }\beta <\mu \Rightarrow \left(\bar{\gamma }\alpha +\bar{\gamma }\bar{\beta }\beta \right)/2<\left(\bar{\gamma }\alpha +\bar{\gamma }\mu \right)/2$$

Considering all the variables are in the range of (0, 1), then $(\bar{\gamma }\alpha +\bar{\gamma }\bar{\beta }\beta )/2$$<(\bar{\gamma }\alpha +\bar{\gamma }\mu )/2$, which means that the successful eavesdropping probability in the proposed system is less than the successful eavesdropping probability in the distributed system proposed in [11].

To sum up, the value of a successful eavesdropping probability in the proposed system is less than the considered two systems’ values. Thus, the private individual data are better protected in the proposed system.

## 8. Conclusions

In recent years, several researchers have agreed that the combination of blockchain and IoT generates a peer-to-peer system, in which peers interact in an untrustless and auditable manner. However, few proposed solutions have dealt with taking advantage of this technology in order to preserve the individuals’ IoT data privacy from an end-to-end perspective. For this reason, we have proposed PrivDA, an end-to-end privacy-preserving IoT data aggregation scheme based on both blockchain and homomorphic encryption technologies. Blockchain acted as a distributed data storage that eliminated the single point of trust issue and the proposed smart contract acted as a data aggregation controller. On the top of the blockchain, the homomorphic encryption technology is used to overcome the raw data disclosure problem, the single point of trust issue, and enable computation over encrypted IoT data. Moreover, we have realized several performance experiments in order to demonstrate the efficiency of the proposed scheme. Then, both computational complexity and communication cost are analyzed. The obtained results showed that our proposal protected the IoT data better than the considered solutions.

Note that use of the blockchain leads to a storage overhead cost. In future work, we plan to store only the newer blocks in order to overcome this issue. Indeed, the consumer does not require storing all the blockchain for a long term. Thus, it can only save the hash of the previous blocks and not the entire blocks to keep the blockchain immutable. Moreover, we intend to incorporate the differential privacy technique in the proposed scheme to enhance the individual’s privacy. The idea behind this is to add noise to the group members’ participation to prevent the consumer from inferring extra information when a group member leaves one group. The impact of the added noise on the data accuracy and the blockchain size needs to be carefully investigated.

## Figures and Tables

**Table 1:** Notations.

Acronym	Descriptions
$SD_{i}$	The *i*-th smart device
$Pk_{SD}$	The SD’s public key
$Sk_{SD}$	The SD’s private key
$Pk_{Agg}$	The aggregator’s public key
$Sk_{Agg}$	The aggregator’s private key
$Pk_{Pai}$	Public key of Paillier cryptosystem
$Sk_{Pai}$	Private key of Paillier cryptosystem
Enc(M,k)	Encrypting a message *M* using the key *k*
Dec(M,k)	Decrypting a message *M* using the key *k*
Sign(M,k)	Signing a message *M* using the key *k*
$participation_{i}$	The encrypted participation of the *i*-th smart device using the *Pk_Pai_*
$encParticipation_{i}$	The encrypted participation of the *i*-th smart device using the aggregator’s public key
$hash_{encP}$	The hash of an encrypted participation
$signature_{hash}$	The signature of an encrypted participation hash
computed_hash	The computed hash from the received data
computed_identity	The computed identity from the received hash and signature
encRequestResult	The encrypted sum of all the encrypted participations in one group
request_result	The decrypted result of data aggregation process

**Table 2:** Computation complexity.

Entity	Operations	Computation Complexity
Producer	Data encryption	$C_{m}+2C_{e}+C_{h}$
	Signature generation	
Aggregator	Sender’s identity and	
	data integrity verification	$\left(k+1\right)*\left(C_{h}+C_{m}\right)$
	Data Aggregation	
	Signature generation	
Consumer	Sender’s identity and	
	data integrity verification	$C_{h}+C_{m}+C_{e}$
	Data decryption	

**Table 3:** Communication cost comparison.

	Our Scheme	[7]	[4]	[9]
**Producer-To-Aggregator**	2048	2144	3136	5344
**Aggregator-To-Consumer**	2048	2144	4160	3200
**Total** (*let k the number of producers*)	k × 2048 + 2048	k × 2144 + 2144	k × 3136 + 4160	k × 5344 + 3200

**Table 4:** Used variables in the comparison.

	Consumer	Aggregator	Producer
Hacking into/Manipulating data in	γ	β	α
Gaining private key of	$\bar{\gamma }$	$\bar{\beta }$	$\bar{\alpha }$

**Table 5:** Comparison of eavesdropping probability of different system components.

		Centralized System	Distributed System [11]	Proposed PrivDA
	data type	N/A	individual(ciphertext)	individual(ciphertext)
Producer	eavesdropping probability	-	$(\bar{\gamma }\alpha +\bar{\gamma }\mu )/2$	$\bar{\gamma }\alpha$
	data type	individual(ciphertext)	aggregated(ciphertext)	individual(ciphertext)
Producer-Aggregator	eavesdropping probability	N/A	N/A	N/A
	data type	individual(plaintext)	aggregated(ciphertext)	indi/aggr(ciphertext)
Aggregator	eavesdropping probability	β	N/A	$\bar{\gamma }\bar{\beta }\beta$
	data type	individual(ciphertext)	aggregated(ciphertext)	aggregated(ciphertext)
Aggregator-Consumer	eavesdropping probability	N/A	N/A	N/A
	data type	individual(plaintext)	aggregated(plaintext)	aggregated(plaintext)
Consumer	eavesdropping probability	γ	N/A	N/A
